# Beyond Toxin Transport: Novel Role of ABC Transporter for Enzymatic Machinery of Cereulide NRPS Assembly Line

**DOI:** 10.1128/mBio.01577-20

**Published:** 2020-09-29

**Authors:** A. Gacek-Matthews, Z. Chromiková, M. Sulyok, G. Lücking, I. Barák, M. Ehling-Schulz

**Affiliations:** a Institute of Microbiology, Vetmeduni Vienna, Vienna, Austria; b Institute of Molecular Biology, Slovak Academy of Sciences, Bratislava, Slovakia; c Institute for Bioanalytics and Agro-Metabolomics, Department IFA-Tulln, University of Natural Resources and Life Sciences Vienna (BOKU), Vienna, Austria; d ZIEL Institute for Food and Health, Technische Universität München, Freising, Germany; University of Maryland School of Medicine

**Keywords:** *Bacillus cereus*, cereulide, ABC transporter, nonribosomal peptide synthetases (NRPS), bacterial adenylate cyclase-based two-hybrid system (BACTH)

## Abstract

Nonribosomal peptide synthetases (NRPSs) and polyketide synthetases (PKSs) play a pivotal role in the production of bioactive natural products, such as antibiotics and cytotoxins. Despite biomedical and pharmaceutical importance, the molecular mechanisms and architectures of these multimodular enzyme complexes are not fully understood. Here, we report on an ABC transporter that forms a vital part of the nonribosomal peptide biosynthetic machinery. Emetic Bacillus cereus produces the highly potent, mitochondrial active nonribosomal depsipeptide cereulide, synthesized by the NRPS Ces. The *ces* gene locus includes, next to the structural *cesAB* genes, a putative ABC transporter, designated *cesCD*. Our study demonstrates that tethering of CesAB synthetase to the cell membrane by CesCD is critical for peptide assembly. *In vivo* studies revealed that CesAB colocalizes with CesCD on the cell membrane, suggesting direct involvement of this ABC transporter in the biosynthesis of a nonribosomal peptide. Mutation of *cesCD*, disrupting the assembly of the CesCD complex, resulted in decreased interaction with CesAB and, as a consequence, negatively affected cereulide biosynthesis. Specific domains within CesAB synthetase interacting with CesC were identified. Furthermore, we demonstrated that the structurally similar BerAB transporter from Bacillus thuringiensis complements CesCD function in cereulide biosynthesis, suggesting that the direct involvement of ABC transporter in secondary metabolite biosynthesis could be a widespread mechanism. In summary, our study revealed a novel, noncanonical function for ABC transporter, which is essential for megaenzyme functionality of NRPS. The new insights into natural product biosynthesis gained may facilitate the discovery of new metabolites with bioactive potential.

## Abstract (precis)

This study revealed a novel, potentially conserved mechanism involved in the biosynthesis of microbial natural products, exemplified by the mitochondrial active depsipeptide cereulide. Similar to other bioactive substances, such as the last-resort antibiotics vancomycin and daptomycin, the antitumor drug cryptophycin or the cholesterol-lowering agent lovastatin, cereulide is synthesized nonribosomally by multienzyme machinery, requiring the concerted actions of multiple proteins to ensure correct product assembly. Given the importance of microbial secondary metabolites in human and veterinary medicine, it is critical to understand how these processes are orchestrated within the host cells. By revealing that tethering of a biosynthetic enzyme to the cell membrane by an ABC transporter is essential for nonribosomal peptide production, our study provides novel insights into synthesis of microbial secondary metabolites, which could contribute to isolation of novel compounds from cryptic secondary metabolite clusters or improve the yield of produced pharmaceuticals.

## INTRODUCTION

Nonribosomal peptide synthetases (NRPSs) are multimodular bacterial and fungal megaenzymes responsible for the synthesis of various bioactive natural compounds, such as antibiotics (e.g., penicillin and vancomycin), antitumor drugs (e.g., bleomycins and cryptophycins), immunosuppressants (e.g., cyclosporine), siderophores (e.g., enterobactin), and toxins (e.g., microcystins and cereulide) (1–5). Thus, it is of utmost importance to understand the mechanism of biosynthesis of these *in vivo* enzymatic machineries, which are organized in modules. Each NRPS module, responsible for recognition, activation, and the condensation of selected monomers, comprise at least an adenylation domain, a peptidyl carrier protein domain, and a condensation domain (A, PCP, and C domains, respectively) (6, 7). A domains act as selective “gatekeepers” recognizing and activating the precursors of the growing peptide, while PCP domains interact with A domains to form thioester intermediates and function as a peptide shuttle, transferring the intermediates between the A and C domains (8). The latter domains catalyze peptide bond formation between two substrates from the adjacent modules. In addition to these essential domains, NRPSs can harbor further domains, such as epimerases, methylases, and ketoreductases (E, M, and KR domains), increasing the biodiversity of peptide products (7). The last module of many NRPSs contains a terminal thioesterase (TE) domain, which catalyzes the release of the final product (9). Although considerable progress has been made in deciphering the structure and function of NRPS domains, it remains elusive how these large, highly dynamic and flexible multienzyme machineries are stabilized and organized in host cells to orchestrate NRPS domains and ensure correct product synthesis.

Notably, NRPS gene clusters frequently harbor ATP-binding cassette (ABC) transporters, which have been reported to be involved in peptide export and/or self-resistance (10). ABC transporters are a superfamily of proteins found in all kingdoms of life that are characterized by conserved structures, typically consisting of two transmembrane domains (TMDs) and two nucleotide binding domains (NBDs) involved in substrate and ATP binding, respectively (11). Apart from the well-established role of ABC transporters in drug resistance and secondary metabolite export (12), there are some indications that ABC transporters might also be involved in the biosynthesis of nonribosomally assembled secondary metabolites, such as toxins, antibiotics, and siderophores (13–15), but the underlying mechanisms remain unclear.

Bacillus cereus is an opportunistic human pathogen that causes two distinct types of food poisoning. Various enterotoxins have been linked to the diarrheal form of the illness, while the emetic form of the illness is caused by depsipeptide toxin cereulide (16, 17). In contrast to the ribosomally encoded enterotoxins that are broadly distributed among the members of the B. cereus group, the potential for the production of the nonribosomally assembled depsipeptide cereulide is restricted to genetically closely related B. cereus strains (18, 19). In emetic Bacillus cereus a putative ABC transporter has been identified that is part of the genetic locus encoding the cereulide (Ces) NRPS responsible for the assembly of the highly bioactive and potent mitochondrial toxin cereulide (20). Ingestion of food contaminated with cereulide provokes emesis and occasionally leads to rhabdomyolysis, liver damage, and serious multiorgan failure, resulting in occasional fatalities (21–24). The cereulide synthetase *ces* genes are organized in an operon localized on the pCER270 virulence megaplasmid, which shares its backbone with the Bacillus anthracis toxin plasmid pX01 (20, 25). The *ces* operon includes a phosphopanteinyl transferase gene (*cesP*) involved in the activation of the NRPS, a putative type II thioesterase gene (*cesT*), the structural NRPS genes (*cesAB*), and an ABC transporter (*cesCD*) (20). As shown previously, expression of the *cesPTABCD* genes, which are polycistronically transcribed by a central promoter upstream of *cesP* (26), is a tightly regulated process that involves different realms of regulation (26–30). Deletion of *cesCD* disables cereulide biosynthesis, though transcription and translation of *cesAB* synthetase are not affected in the mutated strain (31), suggesting that the ABC transporter CesCD plays an essential role in cereulide biosynthesis.

To elucidate the role of ABC transporters in nonribosomal peptide biosynthesis, we investigated possible interactions between the CesCD transporter and selected domains of the CesAB synthetase *in vitro* and *in vivo*. These studies, which provided evidence for direct interaction of the ABC transporter CesCD and the CesAB synthetase, were complemented by *in vivo* colocalization experiments, using Bacillus subtilis as a heterologous host. The genetic and biochemical data presented shed light on a so far poorly understood, yet potentially critical, aspect of secondary metabolite biosynthesis.

## RESULTS

### Interaction of CesAB NRPS with CesC ATPase identified by bacterial two-hybrid screen.

**FIG 1:**
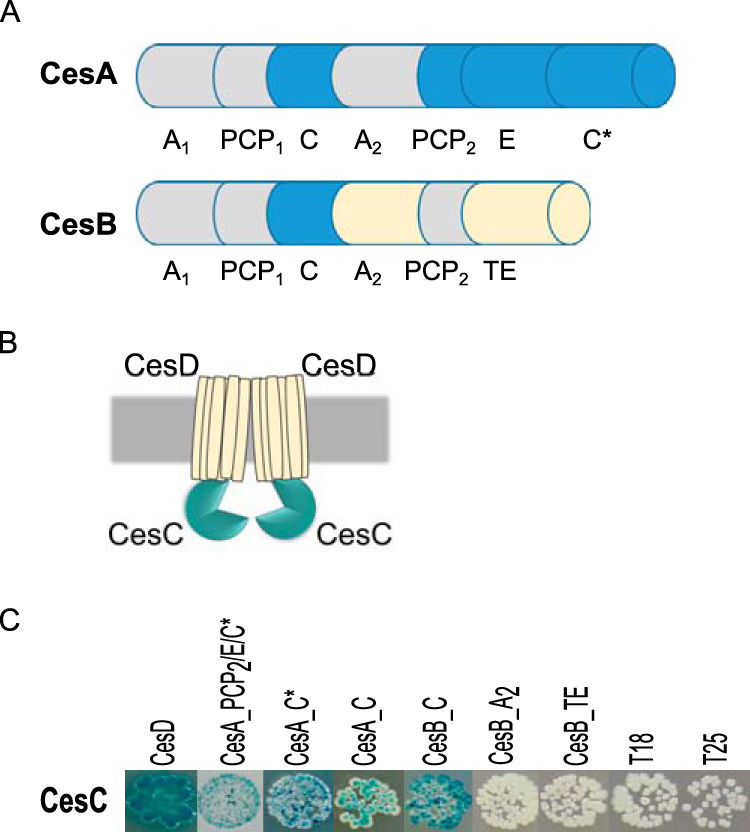
Bacterial two-hybrid (BACTH) screen of CesAB and CesCD interacting domains. (A) Sketch with the designated domains of CesAB: adenylation (A), peptidyl carrier protein (PCP), condensation (C or C*), epimerization (E), and thioesterase (TE). Domains of CesAB interacting with CesC are shown in blue, domains not interacting with CesC in X-Gal assay are shown in yellow, and regions not tested are indicated in gray. No interaction between CesD and CesAB was detected (data not shown). (B) Schematic representation of ABC transporter CesCD, consisting of two nucleotide binding domains (CesC) and two membrane spanning domains (CesD). (C) BACTH assay to screen for interactions between with CesC and CesD as well as CesC and selected domains of CesAB using LB X-Gal/IPTG agar.

**FIG 2:**
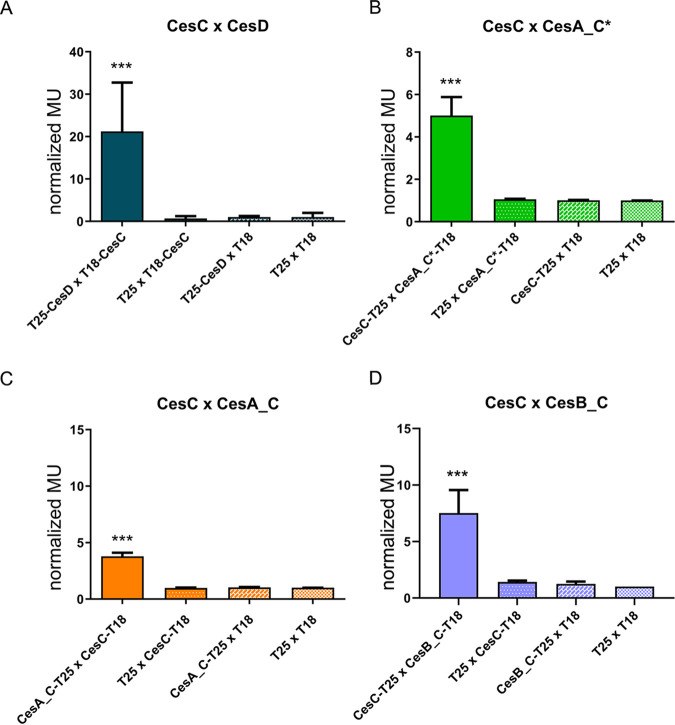
BACTH analysis of binary interactions between CesC and CesD as well as CesC and C condensation (C) domains from CesAB. Normalized β-galactosidase activity of the cells coexpressing T18-CesC and T25-CesD (A) CesC-T25 and CesA_C*-T18 (B), CesC-T18 and CesA_C-T25 (C), and CesC-T18 and CesB_C-T25 (D) expressed in Miller units (MU) is shown. The mean values from each experiment were normalized to the negative-control values (BTH101 cells coexpressing only T18 and T25 subunits of adenylate cyclase). The values of specific negative controls (only one subunit of adenylate cyclase fused with the respective target; the second subunit remained free) are also shown. Each experiment was performed at least three times independently. Error bars represent standard deviation from the mean value. ***, *P* < 0.0001 as determined by *t* test (two-sample assuming equal variances).

In order to identify interactions between Ces NRPS and the ABC transporter, we employed the bacterial adenylate cyclase-based two-hybrid system (BACTH), which is an *in vivo* genetic approach based on reconstitution of the Bordetella pertussis adenylate cyclase (Cya) enzyme if proteins of interest interact (32). Ces NRPS, like other enzymes of this class, is a large multimodular protein complex, consisting of CesA (375 kDa) and CesB (300 kDa) subunits (20). Due to the large size of this complex, it is difficult to assess interactions between CesAB and CesCD directly. Consequently, we selected candidate domains within CesAB for BACTH screening (Fig. 1A). We tested all three condensation domains of the complex (the C and C* domains of CesA and the C domain of CesB), as well as one adenylation domain (A_2_) and the thioesterase domain (TE) of CesB, for potential interactions with CesC. In addition, the C-terminal fragment of CesA, comprising the peptidyl carrier PCP_2_ and the epimerization (E) and C* domains, was tested for interaction with CesC and CesD. Since CesCD is a putative ABC transporter consisting of ATPase CesC and transmembrane protein CesD (Fig. 1B), we also checked the interaction between these two proteins as a proof of concept. For this purpose, selected *ces* domains as well as *cesC* and *cesD* were subcloned into BACTH vectors pUT18C and pKT25. The new constructs expressed respective CesA/CesB domains, CesC and CesD with N- and C-terminal fusions to the B. pertussis adenylate cyclase domains T18 and T25. Combinations of recombinant plasmids were cotransformed into the Escherichia coli BTH101 reporter strain, deficient in adenylate cyclase gene (*cya*). When an interaction between the proteins of interest occurs, T18 and T25 are reunited, reconstituting a catalytically functional adenylate cyclase that boosts the production of cAMP in the E. coli *cya*-deficient host, resulting in transcription activation of reporter genes under the control of a CAP/cAMP promoter, such as a reporter gene encoding the β-galactosidase. The activity of β-galactosidase can be tested using isopropyl-β-d-thiogalactopyranoside/5-bromo-4-chloro-3-indolyl-β-d-galactopyranoside (IPTG/X-Gal) plates or quantified using the liquid Miller assay (33). An overview of tested proteins and domains is provided in Table S1 in the supplemental material, and results of the BACTH assay are shown in Fig. 1 and 2 and Fig. S1.

10.1128/mBio.01577-20.1 FIG S1 Control BACTH assay testing B. subtilis MinD putative interaction with CesA. Shown are positive interaction between MinD-MinD and tested interactions MinD- CesA_PCP_2_/E/C* and MinD-CesA_C* and the respective controls plated on LB X-Gal/IPTG agar. Download FIG S1, EPS file, 0.3 MB. Copyright © 2020 Gacek-Matthews et al. 2020 Gacek-Matthews et al. This content is distributed under the terms of the Creative Commons Attribution 4.0 International license.

10.1128/mBio.01577-20.3 TABLE S1 Proteins and domains tested in the BACTH screen. Amino acid sections are given as start and end amino acid positions with respect to methionine +1. Domain abbreviations refer to the condensation (C or C*), the peptidyl carrier protein (PCP), the epimerization (E), the adenylation (A_2_), and the thioesterase (TE) domains. na, not applicable. Download Table S1, EPS file, 0.6 MB. Copyright © 2020 Gacek-Matthews et al. 2020 Gacek-Matthews et al. This content is distributed under the terms of the Creative Commons Attribution 4.0 International license.

As expected, the coexpression of CesC and CesD with N- and C-terminal fusions to the *cya* fragments T18 and T25, respectively, resulted in the formation of blue colonies on X-Gal/IPTG medium, indicating a positive interaction between CesC and CesD (Fig. 1C). An interaction was also observed between CesC and the second CesA module including PCP_2_, E, and C* (cesA_PCP_2_/E/C*) (Fig. 1C) but not between *cesD* and cesA_PCP_2_/E/C* (data not shown). Notably, the BACTH assay revealed an interaction between CesC and all C domains from CesA and CesB, while no interaction between CesC and the A_2_ domain of CesB (CesB_A_2_) or the TE domain (CesB_TE) was observed (Fig. 1C). Overall, these results indicate a specific interaction between the putative ATPase CesC and the Ces NRPS. To ensure the specificity of the interaction between CesC and Ces NRPS, additional BACTH control assays were performed by using cells coexpressing T18/T25-CesA_PCP_2_/E/C* with T18/T25 tags fused to MinD from B. subtilis, a protein unrelated to cereulide biosynthesis. MinD, which is a vital part of the B. subtilis division site selection system, is an ATPase that binds reversibly to the cell membrane and recruits the MinC protein, an inhibitor of tubulin-like FtsZ assembly (34). No detectable BACTH signal were observed in BTH101 cells coexpressing T18/T25-CesA_PCP_2_/E/C* and T18/T25- MinD (Fig. S1).

To evaluate the strength of the interaction between two hybrid proteins, β-galactosidase enzymatic activity in bacterial extracts was quantified (Fig. 2). As expected, strong β-galactosidase activity, approximately 20-fold compared to that of the negative control (*P* < 0.0001), was detected between the subunits CesC and CesD of the putative ABC transporter. Furthermore, the interactions between CesC and the C domains from CesA and CesB resulted in a significant increase in β-galactosidase activity compared to that of the negative controls (*P* < 0.0001).

### C-terminal condensation domain of CesA interacts with CesC.

**FIG 3:**
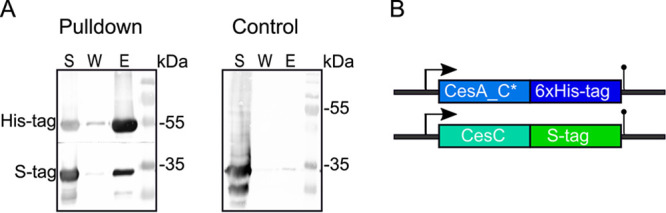
Interactions of CesC and CesA_C* tested by hexahistidine pulldown. CesA_C*-6×His tag pulls down CesC-S tag from E. coli cell lysates on an Ni^2+^ column. (A) Soluble fractions (S) from E. coli cells expressing fusion proteins as well as the final wash fraction (W) and eluent (E) were tested by multiplex Western blotting using anti-His tag and anti-S tag antibodies. As a control for unspecific binding of CesC-S tag fusion protein to the Ni^2+^ column, lysates from the cells expressing only CesC-S tag were applied to the column, washed, and eluted with imidazole (control panel). (B) Graphical representation of the labeling strategy. Both *cesC* and the DNA sequence coding for the C* condensation domain of *cesA* were cloned in frame with S tag and 6×His tag, respectively, in pET Duet expression vector.

In order to verify the interaction between the Ces NRPS (CesAB) and the ATPase CesC observed in the BACTH screen, CesA_C*, suggested previously to play a central role in cereulide assembly (35), was fused to a 6×His tag, while CesC was fused to an S tag. Extracts from E. coli cells overexpressing both fusion proteins (CesA_C* 6×His tag and CesC S tag) were loaded onto an Ni^2+^ column. After several washing steps followed by elution, the fusion proteins were subsequently detected by multiplex Western blotting, using anti-His tag and anti-S tag antibodies. As shown in Fig. 3 and Fig. S2, CesA_C*-6×His tag protein pulled down the CesC-S tag protein, indicating interaction between the C* domain of CesA and the ATPase CesC. To exclude unspecific binding of the CesC-S tag protein, extracts from the cells expressing only CesC-S tag were loaded onto the Ni^2+^ column as a control, which indeed resulted in hardly any detectable protein in the eluate (Fig. 3, control). In summary, the pulldown experiment confirmed specific interaction between the C-terminal condensation domain C* of CesA and CesC *in vitro*.

10.1128/mBio.01577-20.2 FIG S2 Pulldown of CesC by C-terminal condensation domain of CesA. (A) Visualization of the proteins with 2,2,2-trichloroethanol after transfer onto a nitrocellulose membrane. (B) Multiplex Western blot of samples from pulldown experiments. CesA_C*_6×His tag pulled down CesC-S tag (pulldown panel) shown as composite blot image. Unspecific binding of CesC-S to the Ni^2+^ column was tested using lysates of cells expressing only CesC-S tag (control panel). The signals recorded in the different channels are shown as multichannel overlays: chemiluminescence for the CesA_C*-6×His tag fusion protein (indicated in blue), fluorescence for the CesC-S tag fusion protein (indicated in green), and colorimetry for the protein size marker (indicated in red). Signals were recorded simultaneously in the different channels from the same blot, using a multichannel ChemiDoc MP imaging system (Bio-Rad, CA). S, soluble fractions (input); W, final wash; E, eluent. Download FIG S2, EPS file, 2.1 MB. Copyright © 2020 Gacek-Matthews et al. 2020 Gacek-Matthews et al. This content is distributed under the terms of the Creative Commons Attribution 4.0 International license.

### Tethering of CesA_C* domain to the cell membrane is CesCD dependent.

CesCD is a predicted bacterial ABC transporter consisting of the ATPase CesC and the transmembrane protein CesD (Fig. 1B). Consequently, the interaction partner of CesC is thought to be attached to the cell membrane. In order to test this hypothesis, cesA_C*-mNeon green (mNG) fusion was constructed using double-joint PCR (DJ PCR) (36), cloned into the pDG1664 suicide vector, and integrated into the homoserine kinase gene *thrB* in B. subtilis 168. *cesC* was C-terminally labeled with mScarlet (mSc) and, together with unlabeled *cesD*, cloned into pKAM241 suicide vector and integrated into the alpha-amylase locus *amyE* of B. subtilis 168. The new strain was designated the Bs_cesC-mSc_cesD/cesA_C*-mNG strain. As a control, strains with cesA_C*-mNG integrated into *thrB* together with either only *cesC*-mSc or unlabeled *cesD* integrated into *amyE* were constructed. The recombinant control strains were designated the *Bs_cesC*-mSc/cesA_C*-mNG and Bs_cesD/cesA_C*-mNG strains, respectively. By using a *cesP* promoter-*lux* reporter fusion inserted into the B. subtilis 168 *amyE* locus, we recently showed that the B. cereus *cesP* promoter is active in B. subtilis (A. Gacek-Matthews, J. Altenbuchner, and M. Ehling-Schulz, unpublished data). Therefore, all gene fusions were expressed from the native B. cereus *cesP* promoter. The full genotypes of the strains are provided in Table 1.

**TABLE 1:** Bacterial strains used in this study

Strain	Genotype or relevant characteristics	Source or reference
E. coli		
TOP10	Cloning host	Invitrogen
INV110	Methylase-deficient cloning host	Invitrogen
BTH101	Adenylate cyclase deficient (*cya*) reporter strain used in BACTH assay	Euromedex
BL21(DE3)	Expression of the proteins for the pulldown assay	Novagen
Tuner(DE3)	Expression of the proteins for the pulldown assay *lacZY* deletion mutant	Novagen
B. cereus		
F4810/72 (AH187)	Emetic reference strain	3
F48ΔcesCD	*ΔcesCD*::Spc^r^	31
F48ΔpCER270	pCER270 plasmid-cured strain	31
F48ΔcesCD^cesCD^	*ΔcesCD*::Spc^r^ *pAD123_PcesP_cesCD*	31
F48ΔcesCD^berAB^	*ΔcesCD*::Spc^r^ *pWH1520_Pxyl berAB(Bt)*; Tet^r^	This study
F48ΔcesCD^cesCDΔWalkerA^	*ΔcesCD*::Spc^r^ *pAD123_PcesP_cesCD*^Δ^*^walkerA^*; Cm^r^	This study
F48ΔcesCD^cesCD_K40M^	*ΔcesCD*::Spc^r^ *pAD123_PcesP_cesCD^K40M^*; Cm^r^	This study
F48ΔcesCD^cesCD_V29T/F30Y^	*ΔcesCD*::Spc^r^ *pAD123_PcesP_cesCD^V29T/F30Y^*; Cm^r^	This study
B. subtilis		
168	Reference strain	66
Bs_cesCD/cesA_C*	*amyE*::[*PcesP_cesC_mScarlett_cesD_T7t*; Spc^r^], *thrC*::[PcesP_cesA-C*_mNeon Green; Erm^r^]	This study
Bs_cesC/cesA_C*	*amyE*::[*PcesP_cesC_mScarlett_T7t*; Spc^r^], *thrC*::[PcesP_cesA-C*_mNeon Green; Erm^r^]	This study
Bs_cesD/cesA_C*	*amyE::*[*PcesP_cesD_T7t*; Spc^r^], *thrC*::[PcesP_cesA-C*_mNeon Green; Erm^r^]	This study
B. thuringiensis 407	Reference strain	67

**FIG 4:**
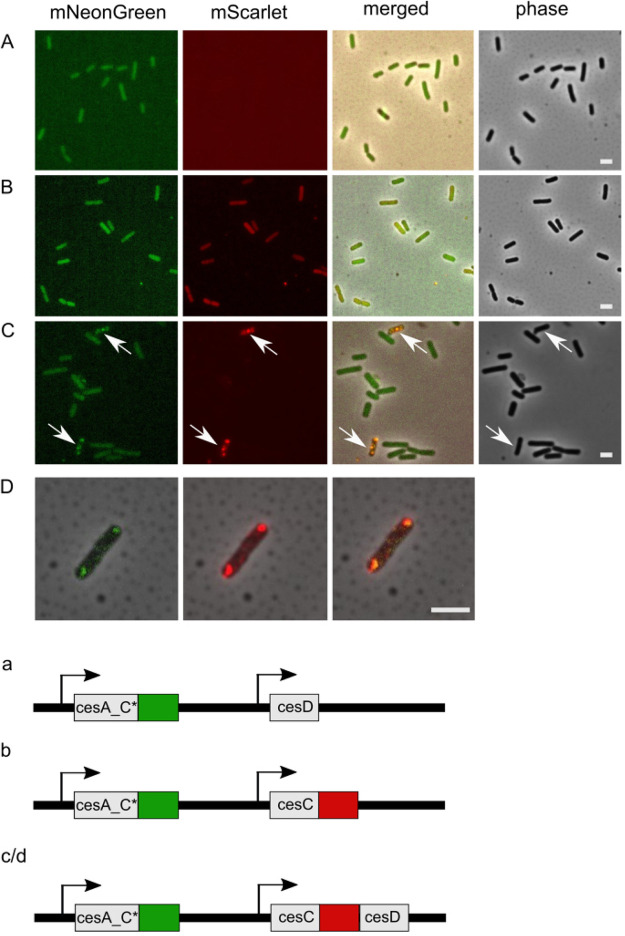
Localization of Ces proteins in B. subtilis 168. (A) Localization of CesA_C*-mNG in the absence of CesC but in the presence of unlabeled CesD (Bs_cesD/cesA_C*-*mNG*). (B) Localization of CesA_C*-mNG in the presence of CesC-mSc but absence of CesD (Bs_cesC-mSc/cesA_C*-mNG). (C) Colocalization of CesA_C*-mNG and CesC-mSc upon the presence of CesD (Bs_cesC-mSc_D/cesA_C*). (D) Merged images with phase contrast showing the colocalization of CesA_C*-mNG and CesC-mSc in the presence of CesD for one single cell. Labeling as in panels A to C. Panels a, b, and c/d show the *ces* constructs used to design the corresponding strains shown in panels A to D. mNG fluorophore is indicated in green, while mSc is indicated in red. The native *cesP* promoter (26), indicated by arrows, was used for expression. Strains were grown for 20 h at 30°C on MYP agar. Scale bars, 2 μm.

As revealed by fluorescence microscopy (Fig. 4), coexpression of cesA_C*-mNG with gene fusion *cesC*-*mSc_cesD* in the *Bs_cesC-mSc*_*cesD*/cesA_C*-mNG strain led to the formation of membrane-localized foci. In contrast, in the recombinant B. subtilis Bs_cesD/cesA_C*-mNG strain, CesA_C*-mNG was localized in the cytosol. Similarly, when cesA_C*-mNG was coexpressed with *cesC*-mSc (*Bs_cesC*-*mSc*/cesA_C*-mNG), both proteins were detected in the cytosol. There were fewer cells (around 10 from 100 inspected) expressing CesCD_mSc than expressing CesA_C*_mNG, suggesting that CesCD might be toxic for B. subtilis 168. In summary, our *in vivo* colocalization studies showed that CesCD ropes CesA_C* to the cell membrane, and if either CesC or CesD is lacking, CesA_C* is delocalized and found to be diffuse throughout the cytosol.

### Deletion or point mutation of Walker A domain of *cesC* disrupts the interaction with CesA_C* and blocks cereulide biosynthesis.

**FIG 5:**
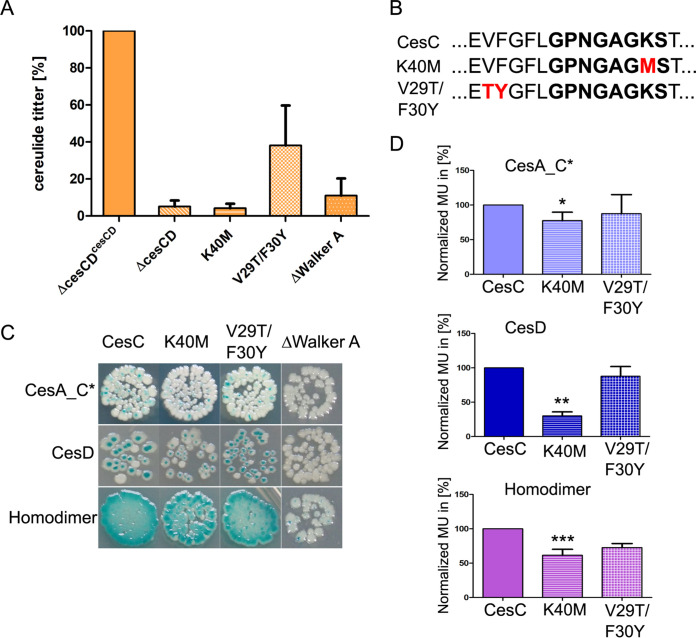
Mutations of *cesC* alter the self-interaction and interaction with CesAB and disable cereulide biosynthesis. (A) HPLC-MS/MS quantification of cereulide in the strains with the mutated versions of cesC (F48ΔcesCD^cesCDΔWalkerA^, F48ΔcesCD^cesCD_K40M^, and F48ΔcesCD^cesCD_V29T/F30Y^) and the *ΔcesCD* mutant. F48ΔcesCD^cesCD^ served as a reference. (B) Fragment of CesC amino acid sequence comprising the Walker A motif, which is indicated in bold. The respective mutations K40M and V29F/F30Y are shown in red. The 33-aa deletion spanning the Walker A motif is too large to illustrate here. (C) BACTH assay analyzing the impact of mutations in CesC (CesC_K40M, CesC_V29F/F30Y, and CesCΔWalker A) on binary interactions with CesA_C* (top row) and with CesD (middle row) as well as on CesC self-interactions (homodimer) (bottom row). (D) β-Galactosidase activity of binary protein combinations, using the mutated versions of CesC depicted in panel B and either CesA_C* (top) or CesD (middle) as a partner. In addition, the effect of mutations on formation of CesC homodimers was tested (bottom). Interactions of CesA_C* and CesD with wild-type CesC served as a reference. Each experiment was performed three times independently. Error bars depict standard deviations from the mean values. ***, *P* < 0.001; **, *P* < 0.01; *, *P* < 0.05 as determined by *t* test (two-sample assuming equal variances).

To investigate whether binding and/or hydrolysis of ATP by CesC is required for the physical contact with CesAB, mutated versions of CesC were constructed and expressed in *trans* together with CesD in an isogenic CesCD null mutant of the emetic B. cereus reference strain F4810/72. As the conserved Walker A motif is known to play a central role in ABC transporters (11), we deleted a 33-amino-acid (aa) region surrounding the conserved Walker A motif in CesC and changed conserved lysine (K) 40 within Walker A (GPNGAGKST) to methionine (K40M) (Fig. 5B). In addition, we exchanged V29T and F30Y, which are located outside Walker A but within the 33-amino-acid region deleted in strain F48ΔcesCD^cesCDΔWalkerA^. All mutated versions of *cesC* were cloned in a pAD123 plasmid, transformed into B. cereus strain F48ΔcesCD, and expressed from the native *cesP* promoter. An overview of the constructed strains is given in Table 1. The new strains, designated F48ΔcesCD^cesCDΔWalkerA^, F48ΔcesCD^cesCD_K40M^, and F48ΔcesCD^cesCD_^*^V29T/F30Y^*, were tested for their cereulide production capacities as described previously (30). The strains F48ΔcesCD^cesCD^ and F48ΔcesCD served as positive and negative controls, respectively. Similar to the case with F48ΔcesCD, the deletion of the 33-aa region spanning Walker A and the exchange of K40M eliminated cereulide production, while the double exchange of V29T and F30Y reduced the cereulide production to approximately 40% of that of the reference strain F48ΔcesCD^cesCD^ (Fig. 5A). In sum, these results suggest a novel type of ABC transporter-NRPS biosynthetic machinery.

To gain further insights into the proposed ABC transporter-NRPS biosynthetic machinery, we tested the constructs from strains F48ΔcesCD^cesCDΔWalkerA^, F48ΔcesCD^cesCD_K40M^, and F48ΔcesCD^cesCD_V29T/F30Y^ in the BACTH screening assay, using CesA_C* and CesD as an interacting partner (Fig. 5C and D). The positive protein-protein interaction between CesC and CesD as well as between CesC and CesA_C* described above (see also Fig. 1) served as controls. In addition, the effect of the mutation in CesC on homodimer formation was tested. The deletion of the 33-aa region including the Walker A motif in CesC aborted the interaction of CesC with both CesA_C* and CesD (Fig. 5C), and exchange of the conserved K40M within Walker A led to significant decrease in β-galactosidase activity of BTH101 cells coexpressing CesC^K40M^ with CesA_C* (approximately 30%; *P* < 0.05) and CesD (approximately 70%; *P* < 0.01) (Fig. 5D). In contrast, the two point mutations V29T and F30Y in CesC outside Walker A did not significantly impact the interactions between CesC and CesA_C* or between CesC and CesD. Interestingly, deletion of the 33-amino-acid region surrounding Walker A not only led to the loss of the interaction with CesA_C* but also disrupted the CesCΔ^WalkerA^-CesCΔ^WalkerA^ homodimer (Fig. 5C and D). Similarly, the exchange of the conserved amino acids K40M and V29T/F30Y showed approximately 40% reduction of β-galactosidase activity in the self-interaction BACTH assay (Fig. 5D). These results emphasize that the interaction between CesC and CesAB as well as the formation of a functional CesCD complex is essential for efficient cereulide biosynthesis.

### Bacillus thuringiensis ABC transporter BerAB interacts with B. cereus Ces NRPS and enables cereulide biosynthesis.

**FIG 6:**
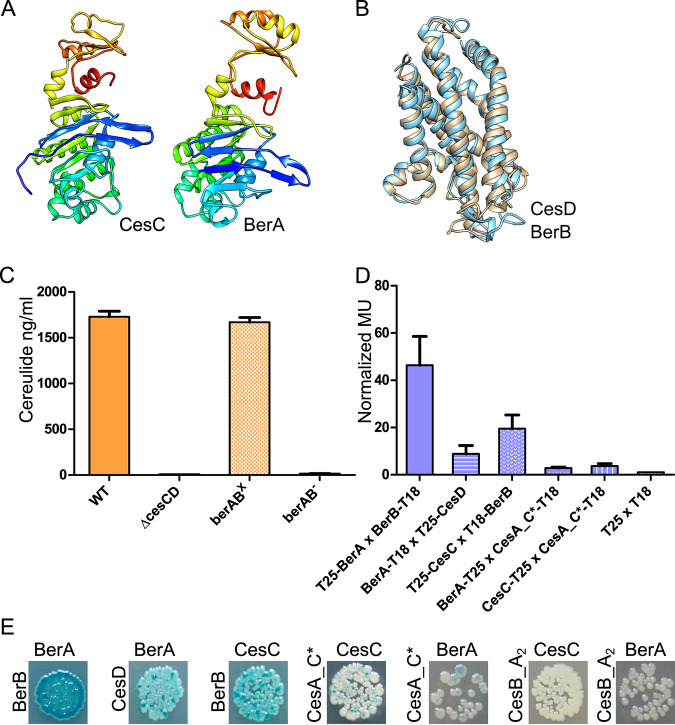
BerAB from B. thuringiensis complements cereulide biosynthesis in F48ΔcesCD. (A) I-TASSER models of CesC and BerA. The structures were drawn with PyMOL. (B) Alignment of I-TASSER-generated ribbon models of CesD (blue) and BerB (brown). (C) Cereulide titer from the total cell extract of the reference strain F4810/72, F48ΔcesCD, and F48ΔcesCD^berAB^ with and without xylose induction (“x” superscript for induction with 0.1% xylose and “−” superscript for no xylose added) quantified by HPLC-MS/MS. (D and E) BACTH experiments analyzing binary interactions between the ABC transporter BerA or CesC and as a partner BerB, CesD, and CesA_C* by β-Gal assay (D) and by using LB X-Gal/IPTG agar (E). Each experiment was performed three times independently. Error bars depict standard deviations from the mean value.

An *in silico* search using BLASTP revealed that the ABC transporter BerAB of B. thuringiensis is the protein most similar to CesCD, with 47.69% amino acid identity and 73.68% similarity between the ATPases CesC and BerA (coding sequence [CDS]: AFV19354.1) as well as 32.08% amino acid identity and 74.62% similarity between the membrane proteins CesD and BerB (CDS: AFV19353.1), respectively. Thus, we employed the I-TASSER platform for protein structure prediction (37) to acquire models for CesC, CesD, BerA, and BerB. As depicted in Fig. 6A and B, the theoretical models for the transmembrane proteins CesD and BerB nearly perfectly match and the models of ATPases CesC and BerA also display high similarities.

To examine whether the role of the ABC transporter in cereulide biosynthesis described here is unique to *cesCD* or whether it is potentially a widespread mechanism, we amplified *berAB* from B. thuringiensis 407, fused it to a xylose-inducible promoter in the expression vector pWH1520, and introduced the constructs into the F4810/72 *ΔcesCD* strain. Induction of *berAB* with 0.1% xylose in the new strain, designated F48ΔcesCD^berAB^, resulted in cereulide production levels comparable to those of the wild type (Fig. 6C). These data clearly demonstrated that the structurally related transporter BerAB complements CesCD function in cereulide biosynthesis, even though B. thuringiensis is not a cereulide producer.

Since BerAB restored cereulide production in F48ΔcesCD to the wild-type (WT) level, it could be assumed that there is also physical contact between CesNRPS CerAB and the ATPase BerA. To test this hypothesis, *berA* and *berB* were cloned into BACTH vectors and interaction studies were carried out as described above. As expected, BerA and BerB showed a very strong interaction (see Fig. 6D and E). However, there were also strong interactions between the ATPase BerA from B. thuringiensis and the membrane protein CesD from B. cereus as well as between the ATPase CesC from B. cereus and the membrane protein BerB from B. thuringiensis. These data indicate that BerAB is similar to CesCD not only in terms of structure but also in terms of function. These findings were corroborated by the results from the BACTH studies of BerA and the condensation domains of the cereulide synthetase CesAB. A positive interaction between BerA and CesA_C* was observed, while no interaction was found for BerA and CesB_A2 (Fig. 6D and E). These results, which are in line with the ones from our interaction studies of CesAB and CesC (see Fig. 1), demonstrated that the described function of the ABC transporter CesCD in nonribosomal peptide biosynthesis could indeed be a widespread mechanism. To test this hypothesis, we used the Minimum Information about a Biosynthetic Gene cluster (MIBiG) repository (38) to search for CesC-homologous ATPases associated with NRPS gene clusters. As shown in Table S4, this search revealed several putative ATPases adjected to NRPS gene clusters, showing between 46% and 32% identity to CesC.

With our work we showed that not only CesCD, encoded within the cereulide operon, provides an essential function in cereulide biosynthesis but also that the structurally similar transporter B. thuringiensis BerAB, *nota bene* not located within any NRPS biosynthetic locus, could functionally complement CesCD by creating a biosynthetic complex with the CesAB cereulide synthetase.

## DISCUSSION

Bacterial multidrug ABC transporters play a vital role in bacterial physiology, controlling processes such as resistance to antimicrobial substances, virulence, and biosynthesis of bioactive natural products. Frequently, transporter inactivation leads to intracellular accumulation of the otherwise excreted metabolites (39, 40). So far, a small number of studies has documented a cessation of toxin synthesis (rather than intracellular toxin accumulation) upon inactivation of respective transporters. For example, transposon mutagenesis leading to inactivation of the ABC transporter ExiT, which forms part of the NRPS gene cluster responsible for nonribosomal synthesis of the siderophore exochelin, resulted in an exochelin-negative phenotype in Mycobacterium smegmatis (15). Similarly, we have shown that deletion of the ABC transporter CesCD, encoded in cereulide synthetase operon in emetic B. cereus (20), resulted in a cereulide-negative phenotype. This suggests that biosynthesis of this nonribosomal peptide was blocked in a hitherto-unknown manner (31). To elucidate the function of the ABC transporter CesCD in cereulide biosynthesis, we investigated if the CesCD transporter interacts with CesAB NRPS and consequently tethers this multimodular enzyme complex to the cell membrane, thereby potentially enabling the adoption of the optimal conformation for depsipeptide assembly. Such allosteric regulation could control the function of cereulide synthetase as well as efficiency of the whole assembly line in B. cereus.

### Novel type of ABC transporter-NRPS biosynthetic machinery, exemplified by Ces NRPS.

Our data convincingly show that an ABC transporter adjacent to a nonribosomal peptide synthetase plays a crucial and direct role in the biosynthesis of a natural product, beyond its canonical transport function. The biosynthesis of cereulide, just as for other nonribosomal peptides, is a multistep, energy-consuming process. Since cereulide is an ionophore leading to membrane depolarization (41, 42), it is tempting to speculate that it would be beneficial for the producing cells to simultaneously orchestrate both the biosynthesis and the export of this metabolite. Subcellular compartmentalization of the metabolic enzymes involved in the primary metabolic processes, such as glycolysis, the tricarboxylic acid cycle, or fatty acid synthesis, is a widespread and highly regulated process (43). In contrast, only a few examples of subcellular compartmentalization in secondary metabolism have been documented. An organelle-like, membrane-associated megacomplex of NRPS/PKS hybrid was described for B. subtilis (44). Furthermore, the temporal membrane localization of the siderophore pyoverdine NRPS in Pseudomonas aeruginosa has been reported in the context of efficient transport of the precursors and the release of the final product to the secretion apparatus (35). As revealed by our study, the ATPase CesC of the ABC transporter CesCD, which is cotranscribed with the structural cereulide synthetase genes *cesAB* (26), specifically interacts with the condensation domains of CesAB. We did not detect any interaction between CesC and the A domains or TE domain of CesB. Since A domains are the catalytical core of each NRPS module responsible for amino, hydroxy, and carboxy acid recognition and activation (3, 45), one could speculate, *a priori*, that a binding pocket will, in order to remain exposed to its substrates, not easily interact in *trans.* In contrast, C domains have been recognized as quite flexible NRPS domains, which can adopt several conformations (46, 47). This observation is fostered by our results, suggesting that the interactions of C domains with CesC, the catalytic part of the ABC transporter, permit CesAB to adopt the optimal conformation for efficient cereulide synthesis. We further demonstrated that CesA_C* colocalizes with CesCD at the cell membrane, while CesA_C* was found to be delocalized and distributed throughout the cytosol in cells not expressing a functional CesCD ABC transporter. As inactivation of either *cesC* or *cesD* led to loss of cereulide production (31), it is tempting to speculate that roping of the cereulide biosynthetic machinery to the cell membrane via CesCD is crucial for cereulide biosynthesis.

Transport of the substrates by ABC transporter is a multistep process. First, nucleotide binding domains (NDBs) undergo dimerization due to the binding of ATP and the substrate. In the second step, ATP is hydrolyzed and the substrate is translocated across the membrane. However, under certain circumstances, ATP binding alone leads to the conformational changes of the transporter to facilitate substrate allocation. In the latter case, ATP hydrolysis and ADP release reset the transporter to the original conformation (48, 49). The NBDs have highly conserved amino acid sequences, such as the Walker A motif (GXXGXG**K**S/T) (lysine in bold) (11), which is also conserved in CesC (Fig. 1B). Within the Walker A motif, the conserved amino acids lysine and glycine have been reported to play a pivotal role for the functionality of the transporter by binding to ATP (50, 51). As revealed by our current work, the Walker A motif in CesC is essential not only for correct CesCD oligomerization but also for the interaction of CesC and CesA_C*, suggesting that it plays an additional role in the NRPS assembly line of cereulide beyond its canonical function. The change of a conserved lysine to methionine (K40M) in the Walker A motif of CesC also influenced the interaction of CesC and CesA_C* and consequently ceased cereulide biosynthesis. In contrast, the cereulide biosynthesis level was only partially affected in the F48ΔcesCD^cesCD_V29T/F30Y^ mutant. The V29 and F30 amino acids, located directly upstream of the conserved Walker A motif, were selected for the mutagenesis because they are conserved between the B. cereus CesCD and B. thuringiensis BerAB transporters (data not shown). Furthermore, these amino acids have not yet been described to play an essential role for the functionality of ABC transporters. It could be speculated that the exchange of V29T/F30Y affects the exact position of Walker A within the structure of the ABC transporter, thereby hindering its functionality. Further structural studies, which are clearly beyond the scope of our current work, will be necessary to fully decipher the molecular interactions between CesCD and CesAB. Nevertheless, the results of the mutagenesis approach and findings from the colocalization studies emphasize that a functional ABC transporter, CesCD, is essential for the correct assembly of the enzymatic CesNRPS machinery directing cereulide biosynthesis.

As demonstrated by BACTH, the structurally similar BerAB transporter from the non-cereulide-producing organism B. thuringiensis, interacts with the C* domain of CesA. This demonstrates a noncanonical function of ABC transporter in natural product synthesis by direct interactions with the NRPS complex. Thus, it could be hypothesized that C domains of CesAB require the interaction with CesCD, in order to adopt optimal conformation for the assembly line, while the absence of CesCD leads to the stalling of CesAB synthetase (47).

Furthermore, heterologous expression of *berAB* in the *ΔcesCD* mutant restored cereulide titer to the wild-type level. These data underscore the crucial role of membrane transporters in both the functionality of a nonribosomal peptide synthetase and the efficiency of subsequent product assembly. Notably, among the 605 NRPS clusters annotated in the MIBiG database (38), we found 14 clusters encoding putative ABC transporters with high homology to CesC. These putative ABC transporters are found in NRPS gene clusters of different bacterial species, including species not related to the B. cereus group, such as Rothia nasimurium, Staphylococcus lugdunensis, Salinispora arenicola, *a Micromonospora* sp., and a *Streptomyces* sp. (Table S4). These findings from our *in silico* analysis foster the hypothesis that ABC transporters could play a crucial role in biosynthesis of natural product beyond their canonical function.

Similarly, an interaction was reported between the MmpL7 permease belonging to the RND (resistance, nodulation, and cell division) family of bacterial transporters and the PpsE polyketide synthase (PKS) involved in the biosynthesis of the lipid phthiocerol dimycocerosate (PDIM) in Mycobacterium tuberculosis (52). However, deletion of *mmpL7* in M. tuberculosis did not alter PDIM synthesis but led to intercellular accumulation of PDIM (52), while the deletion of *cesCD* disabled cereulide production in emetic B. cereus (Fig. 4A) (31). Thus, the interaction between the ABC transporter CesCD and CesAB synthetase is critical not only for coordination of synthesis and transport but also for the functionality of the biosynthetic complex.

In summary, our work revealed novel insights into microbial secondary metabolite biosynthesis and provided evidence for an essential, noncanonical function of ABC transporter in natural product synthesis. These findings are expected to be of high relevance for the understanding of the architecture and cellular organization of NRPS multienzyme machinery and may provide a foundation for future work that could contribute to the discovery of novel bioactive microbial products, which are the major source of antibacterial, anti-infective, and antitumor therapeutics (53). In recent years, great advances have been made in developing tools and techniques to access bacterial and fungal natural products. However, progress has not kept up with demands from a global society that is becoming increasingly reliant on discovery of the novel therapeutics against pan-resistant microbes (54–56). The function of CesCD and BerAB transporters in cereulide biosynthesis reported here may represent a key, widespread mechanism in the assembly line of nonribosomal natural products. Therefore, improved understanding of ABC transporters may indeed allow exploitation of hitherto-cryptic secondary metabolite NRPS clusters (57) for which, despite successful expression of the biosynthetic genes, natural products have not yet been detected.

## MATERIALS AND METHODS

### Bacterial strains, media, and growth conditions.

B. cereus, B. subtilis, and E. coli strains were routinely cultivated on Luria-Bertani (LB) agar or in LB broth unless stated otherwise at 30°C (B. cereus) or at 37°C (B. subtilis and E. coli). Where required, the appropriate antibiotics were added (120 μg/ml of ampicillin, 100 μg/ml of spectinomycin, 50 μg/ml of kanamycin, 10 μg/ml of erythromycin, 5 μg/ml of chloramphenicol) and incubated in 500-ml baffled flasks with rotary shaking at 150 rpm. All strains used are listed in Table 1.

### DNA manipulation and generation of the modified strains.

An overview of plasmids used in this study is provided in Table S2. Oligonucleotides used in this study were synthesized by Eurofins (Ebersberg, Germany) and are listed in Table S3. High-fidelity DNA polymerase (F-530XL; Thermo Fisher Scientific, MA) was used for cloning, restriction enzymes were purchased from New England BioLabs (MA) and Thermo Fisher Scientific, the Wizard SV gel and PCR cleanup system (A9282; Promega, WI) was used for DNA fragment purification, and T4 DNA ligase (EL0011; Thermo Fisher Scientific) was used for ligation. All inserts were proofed by DNA sequencing (LGC Genomics GmbH, Berlin, Germany). E. coli strains were transformed by heat shock, B. cereus by electroporation, and B. subtilis by natural competence transformation as previously described (3, 31, 58).

10.1128/mBio.01577-20.4 TABLE S2 Plasmids used in the study. Download Table S2, XLSX file, 0.01 MB. Copyright © 2020 Gacek-Matthews et al. 2020 Gacek-Matthews et al. This content is distributed under the terms of the Creative Commons Attribution 4.0 International license.

10.1128/mBio.01577-20.5 TABLE S3 List of PCR primers used to create the constructs in this study. Download Table S3, XLSX file, 0.01 MB. Copyright © 2020 Gacek-Matthews et al. 2020 Gacek-Matthews et al. This content is distributed under the terms of the Creative Commons Attribution 4.0 International license.

10.1128/mBio.01577-20.6 TABLE S4 Putative ABC transporters embedded in nonribosomal peptide (NRP) biosynthetic gene clusters (BGC) retrieved from the Minimum Information about a Biosynthetic Gene cluster (MIBiG) repository (https://mibig.secondarymetabolites.org) and known ABC transporters not associated with NRP BGC with sequence identity of at least 32% to CesC, based on BLASTP (https://blast.ncbi.nlm.nih.gov/Blast.cgi) search. PK, polyketide; n.a, not applicable. Download Table S4, PDF file, 0.1 MB. Copyright © 2020 Gacek-Matthews et al. 2020 Gacek-Matthews et al. This content is distributed under the terms of the Creative Commons Attribution 4.0 International license.

### Generation of Walker A deletion and point mutations.

For deletion of the 33-aa region including the Walker A motif of *cesC* (for the amino acid sequence, see Fig. 5B), the pAD123 plasmid containing the *PcesP-cesC-cesD* sequence was used as a template. In the first step the upstream sequence of Walker A including the *cesP* promoter (PcesP) and the *cesC* sequence upstream of Walker A was amplified, while the downstream sequence of Walker A together with *cesD* and the terminator was amplified in the second PCR. Both PCR products were fused using DJ PCR and cloned into the E. coli/B. subtilis shuttle plasmid pAD123 (59). The new plasmid was designated pAD123cesC^ΔWalkerA^. In a similar manner, K40M and V29T/F30Y mutations were inserted by PCR in the reversed primers of the upstream sequences as well as the forward primers of the downstream sequences. As described above, the fragments were joined using DJ PCR and cloned into the pAD123 plasmid, resulting in pAD123cesC^K40M^ and pAD123cesC^V29T/F30Y^, respectively. Subsequently, plasmids were introduced into B. cereus by electroporation and the new strains were designated F48ΔcesCD^cesCDΔWalkerA^, F48ΔcesCD^cesCD_K40M^, and F48ΔcesCD^cesCD_V29T/F30Y^.

### Heterologous expression of BerA in F48ΔcesCD.

To generate a *berAB* overexpression strain, the promoterless *berAB* gene (GenBank accession no. CP003889.1; 3579694 to 3581386) was amplified from the B. thuringiensis 407 strain and cloned into the pWH1520 E. coli/B. cereus shuttle vector carrying a xylose-inducible promoter (60). The plasmid pWH1520^berAB(Bt)^ was introduced into B. cereus F48ΔcesCD by electroporation using tetracycline selection, resulting in F48ΔcesCD^berAB^. Overexpression of BerAB was induced by the addition of 0.1% d-xylose to LB cultures containing 10^3^ CFU/ml of F48ΔcesCD^berAB^.

### Bacterial two-hybrid system and β-galactosidase assay.

*cesC* (NCBI accession no. ABK00528.1), *cesD* (ABK00530.1), and selected domains of *cesA* (YP_001967170.1) and *cesB* (ACJ82785.1) were amplified from the emetic reference strain B. cereus F4810/72 (3) and cloned into pKT25, pKNT25, pUT18, and pUTC18C BACTH expression vectors (catalog no. EUK001; Euromedex, Souffelweyersheim, France). The respective plasmids were introduced into the E. coli BTH101 *cya*-deficient host strain by heat shock transformation. The procedure is based on functional complementation of two subunits of the adenylate cyclase (T18 and T25) fused with the tested interacting partners as previously described (32, 61). For each putative interaction, several combinations were tested as follows: every protein was tagged on the N and C termini as well as by both subunits of adenylate cyclase (T18 and T25; for a detailed description of the cloned domains, the reader is referred to Table S1). Respective combinations were cotransformed in E. coli BTH101 cells and plated on LB X-Gal/IPTG agar or LB IPTG for β-galactosidase assay. The cells were incubated for 24 h at 30°C. Thereafter, the cells were moved to room temperature (RT) for 20 h, transferred to 18°C, and incubated for 20 h. For the β-galactosidase assay, the cells were harvested from the LB-IPTG plates and resuspended in 1 ml of Z buffer (10 mM KCl, 100 mM MgSO_4_, 0.27% β-mercaptoethanol, sodium phosphate buffer [pH 7.0]). Optical density at 600 nm (OD_600_) was adjusted to 0.4 to 0.7, cells were permeabilized using β-mercaptoethanol, SDS, and chloroform, and the enzymatic reaction (at 28°C) was induced by addition of 4 mg/ml of *o*-nitrophenol-β-galactoside (ONPG) in sodium phosphate buffer. The time at which each sample turned yellow was recorded, and the reaction was stopped with 1 M Na_2_CO_3_. OD_420_ and OD_550_ values were measured using a BioSpectrometer basic (Eppendorf, Hamburg, Germany). The β-galactosidase activity was determined according to the method of Miller (61) and expressed in Miller units [MU]. The mean values of β-galactosidase activity from each experiment were normalized by the mean value of the respective negative control (BTH101 cells coexpressing only T18 and T25 subunits of adenylate cyclase). Thus, the indicated positive interactions are expressed as fold change compared to the respective negative control.

### Pulldown assay.

CesC was C-terminally labeled with S tag, while C* domains of CesA were tagged on the C terminus with 6×His tag. Thus, the *cesC* gene and the C* domain of *cesA* were amplified and cloned directly into the pET Duet expression vector in frame with the S and 6×His tags, respectively. The plasmids were transformed into E. coli BL21 or Tuner. Due to problems with solubility and stability of the recombinant proteins, two different expression strains of E. coli were used. CesC-S tag was expressed in the E. coli Tuner strain, while BL21 cells were used for expression of CesA_C*-6×His. Cells were grown to an OD_600_ of 0.3, induced with 0.125 mM IPTG at 18°C overnight, and harvested by centrifugation (20 min and 5,000 rpm). The bacterial pellet from a 100-ml culture of CesA_C*_6×His tag cells (BL21) was resuspended in 2 ml of high-salt solubilization buffer (20 mM Tris-HCl [pH 8], 500 mM NaCl, 1 mM AEBSF, a protease inhibitor including aprotinin, bestatin, E64, leupeptin, pepstatin A, and phenylmethylsulfonyl fluoride [PMSF]) (Thermo Fisher Scientific, MA), and the bacterial pellet from a 50-ml culture of CesC-S tag cells (Tuner) was resuspended in 1 ml of the same buffer. The suspensions were pooled and the mixture was lysed by sonication (15.000 kHz, 25 times for 9 s with a 1-min break and constant cooling), followed by centrifugation for 30 min at 30,000 rpm. The supernatant was used for the pulldown assay and thus applied onto a HisTrap HP column (GE Healthcare, Chicago, IL). The column was washed 9 times with sonication buffer supplemented with 40 mM imidazole, and the captured proteins were eluted with 1 M imidazole. One-milliliter fractions of the column efflux were collected. A total of 100 μl of each fraction was boiled with the equal amount of 2× sample Laemmli buffer, followed by loading of 20 μl of each sample onto 12% SDS-PAGE gels. Proteins were labeled with 2,2,2-trichloroethanol (Sigma, MO) and visualized in a 300-nm UV transilluminator (51). Subsequently, proteins were analyzed by multiplex Western blotting using the Penta-His horseradish peroxidase (HRP) conjugate kit (Qiagen, Hilden, Germany) for detection of CesA_C*_6×His tag by chemiluminescence as well as an S tag monoclonal antibody (Novagen, Darmstadt, Germany) and the StarBright Blue 700 goat anti-mouse secondary antibody (Bio-Rad, CA) for detection of CesC-S tag by fluorescence. Protein sizes were determined using a prestained protein marker detected by colorimetry. Signals from the different channels were recorded simultaneously using a multichannel ChemiDoc MP imaging system (Bio-Rad, CA). To test the unspecific binding of CesC-S tag to the HisTrap HP column, bacterial pellets from a 50-ml culture of CesC-S tag cells (Tuner) were resuspended in 1 ml of high-salt solubilization buffer, sonicated, and centrifuged, and the soluble fraction was loaded onto the column and treated as described above.

### Colocalization experiments.

The promoter of *cesP* and the C* domain of *cesA* were amplified from the reference B. cereus strain F4810/72, while mNeon Green (mNG) was amplified from pET14b_mNeonGreen (a plasmid kindly provided by M. Leake, University of York, UK). All three PCR fragments were merged in DJ PCR with mNG adjusted to the C terminus (36). The fragment containing PcesP-cesA_C*-mNG-T7_terminator_ was cloned into the pDG1664 B. subtilis suicide vector in the region flanked by *thrC* upstream and downstream complementary sequences and comprising the Erm^r^ cassette. Similarly, *PcesP*, *cesC*, and *cesD* were amplified from the reference B. cereus strain F4810/72, while mScarlet was amplified from pET14b_mScarlet. All PCR products were joined in three-step DJ PCR. The fragment containing *PcesP-cesC-mScarlet_stop_-cesD-T7_terminator_* was cloned in the region flanked by *amyE* upstream and downstream complementary sequences as well as comprising the Spr^r^ cassette in the pKAM241 B. subtilis suicide vector (62). Both suicide plasmids pDG1664 PcesP-cesA_C*-mNG-T7_terminator_ and pKAM241 *PcesP-cesC-mScarlet_stop_-cesD-T7_terminator_* were transformed in B. subtilis 168 by natural competence transformation. The new strain was designated Bs_cesCD/cesA_C*. The control strains Bs_cesC/cesA_C* and Bs_cesD/cesA_C* were constructed in the same way. For detailed descriptions of the genotypes and the plasmids and oligonucleotides used in this study, the reader is referred to Table 1 and Tables S2 and S3.

Liquid cultures of B. subtilis Bs_cesC-mSc_D/cesA_C*-mNG, Bs_cesC-mSc/cesA_C*-mNG, and Bs_cesD/cesA_C*-mNG strains containing the fusions of B. cereus *cesP* promoter and selected genes or domains of the cereulide operon with mNeon Green (excitation, 504 nm, and emission, 517 nm) and mScarlet (excitation, 570 nm, and emission, 593 nm) were grown overnight in LB medium at 37°C. The strains were spotted on MYP agar (mannitol-egg yolk-polymyxin) and incubated for 20 h at 30°C. Bacterial colonies were harvested, washed twice with phosphate-buffered saline (PBS), and pelleted by centrifugation for 3 min at 5,000 rpm. The cells were resuspended in PBS, applied to poly-l-lysine-coated slides (Sigma-Aldrich, St. Louis, MO), and analyzed using an Olympus BX63 fluorescence microscope, with cubes U-FBWA for detection of mNeon Green and U-FYW for detection of mScarlet. The microscope was equipped with an Andor Zyla 5.5 sCMOS camera. Olympus CellP imaging software and ImageJ (63) were used for image acquisition and analysis, respectively.

### Cereulide quantification by HPLC-MS/MS.

For quantification of cereulide, 100 ml of LB medium was inoculated with 10^3^ CFU/ml of B. cereus with the appropriate antibiotics in baffled flasks and cells were grown for 24 h at 30°C and 150 rpm. Cereulide was extracted as described previously (30). In brief, bacteria were pelletized by centrifugation (8,000 × *g*, 23°C, and 10 min), and 50 mg of bacterial biomass was resuspended in 1 ml of acetonitrile (99%, high-performance liquid chromatography [HPLC] grade; Carl Roth, Karlsruhe, Germany), followed by incubation for 16 h at RT on a rocking table. Samples were centrifuged, and supernatants were put through Phenex HPLC syringe filters (Phenomenex, Torrance, CA) and directly transferred to the HPLC vials. Cereulide concentrations were determined using a liquid chromatography-tandem mass spectrometry (LC-MS/MS)-based multianalyte method (64). In brief, a QTrap 5500 LC-MS/MS system (Applied Biosystems, Foster City, CA) equipped with a Turbo Ion Spray electrospray ionization (ESI) source was coupled to a 1290 series HPLC System (Agilent, Waldbronn, Germany). Chromatographic separation was performed at 25°C using a Gemini C_18_ column, 150 mm by 4.6-mm inside diameter [i.d.], 5-μm particle size, guarded with a C_18_ 4 mm by 3-mm-i.d. security guard cartridge (Phenomenex). Two methanol/water/acetic acid preparations (10:89:1 and 97:2:1 [vol/vol/vol]), both of which contained 5 mM ammonium acetate, were used as eluents A and B. An injection volume of 5 μl and flow rate of 1 ml/min were used.

### Statistical analysis.

The statistical significance of differences between mean values (α = 0.05) was tested by applying unpaired two-sample Student’s t tests (assuming equal variances) using the software GraphPad Prism 8 (v8.3.0; GraphPad Software, Inc.). Error bars in figures represent standard deviation from the mean value.

### Protein similarity search.

A BLASTP search of bacterial reference genomes in NCBI using the CesCD amino acid sequence from the emetic reference strain F4810/72 (AH187) (3) was performed in order to identify CesCD homologs. Since the results from *in vitro* and *in vivo* interaction studies revealed that the ATPase CesC of the putative ABC transporter CesCD interacts with NRPS CesAB, the MIBiG Database was employed to search for CesC-homologous ABC proteins encoded in other NRPS biosynthetic gene clusters. The Ces cluster nucleotide sequence was therefore submitted to the antiSMASH database (65) and the retrieved CesC protein sequence was subsequently used to search the MIBiG database (38) for NRPS clusters comprising ABC transporters related to CesC. The cutoff for sequence identity between CesC and homologous proteins in NRPS clusters was set to 32%.
