# Supplementary material for: Beyond Toxin Transport: Novel Role of ABC Transporter for Enzymatic Machinery of Cereulide NRPS Assembly Line
Source: mBio. 2020 Sep 29;11(5):e01577-20. doi: 10.1128/mBio.01577-20 (PMC7527721; doi:10.1128/mBio.01577-20)
Supplement: TABLE S4 [file mBio.01577-20-st004.pdf]

**Table S4**. Putative ABC transporters embedded in Non Ribosomal Peptide (NRP) Biosynthetic Gen Clusters (BGC) retrieved from the Minimum Information about a Biosynthetic Gene cluster (MIBiG) repository (https://mibig.secondarymetabolites.org) and known ABC transporters not associated with NRP BGC with sequence identity of at least 32% to CesC, based on BLASTP (https://blast.ncbi.nlm.nih.gov/Blast.cgi) search. Abbreviations: PK: polyketide; n.a: not applicable.

| **MIBiG Protein** | **Description** | **MIBiG Cluster** | **MIBiG Product** | **% ID** |
|---|---|---|---|---|
| WP_000626565.1 | BerA (*Bacillus thuringiensis*) | n.a. | n.a. | 48 |
| ORC16572.1 | ABC | BGC0001341 | putative valinomycin BGC from *Rothia nasimurium* | 46 |
| WP_019032750.1 | ABC | BGC0001331 | BE-435471 BGC (cyclic depsipeptide + polyketide: modular type I) from *Salinispora arenicola* CNR107 | 41 |
| WP_047890617.1 | ABC | BGC0001330 | BE-43547A1 BGC (cyclic depsipeptide + polyketide: modular type I) from *Micromonospora* sp. RV43 | 40 |
| WP_003948917.1 | ABC | BGC0001792 | surugamide A BGC from *Streptomyces albidoflavus* | 39 |
| BAH43868.1 | ABC | BGC0000367 | gramicidin BGC from *Brevibacillus brevis NBRC 100599* | 37 |
| ALV86877.1 | Tlo31 | BGC0001406 | telomycin BGC from *Streptomyces canus* | 36 |
| CAM56766.1 | ExpB | BGC0000354 | friulimicin BGC from *Actinoplanes friuliensis* | 34 |
| QBG38790.1 | Atr29 | BGC0001975 | atratumycin BGC from *Streptomyces atratus* | 34 |
| X73633.1 | TnrB2 (*Streptomyces longisporoflavus*) | n.a. | n.a. | 34 |
| CCB53259.1 | LugE | BGC0001393 | lugdunin BGC from *Staphylococcus lugdunensis N920143* | 33 |
| ADG27347.1 | AcmrA | BGC0000296 | actinomycin D BGC from *Streptomyces anulatus* | 33 |
| BAX64249.1 | Iga8 | BGC0001623 | ishigamide BGC (PK-NRP hybrid) from *Streptomyces sp. MSC090213JE08* | 33 |

| | | | | |
|---|---|---|---|---|
| CAJ34360.1 | TioD | BGC0000445 | thiocoraline BGC from *Micromonospora sp. ML1* | 33 |
| ADG27368.1 | AcmW | BGC0000296 | actinomycin D BGC from *Streptomyces anulatus* | 33 |
| WP_028678143.1 | DrrA | BGC0001228 | retimycin A BGC from *Salinispora arenicola CNT005* | 32 |
| AAD21213.1 | BcrA | BGC0000310 | bacitracin BGC from *Bacillus licheniformis* | 32 |
| CCB53261.1 | LugG | BGC0001393 | lugdunin BGC from *Staphylococcus lugdunensis N920143* | 32 |
| WP_014570413.1 | NisF *(Lactococcus lactis*) | n.a. | n.a. | 32 |
| AAS78451.1 | BcrA (*Enterococcus faecalis*) | n.a. | n.a. | 32 |
